# Temporary “Circuit Breaker” Lockdowns Could Effectively Delay a COVID-19 Second Wave Infection Peak to Early Spring

**DOI:** 10.3389/fpubh.2020.614945

**Published:** 2020-12-07

**Authors:** Thomas Rawson, Chris Huntingford, Michael B. Bonsall

**Affiliations:** ^1^Mathematical Ecology Research Group, Department of Zoology, University of Oxford, Oxford, United Kingdom; ^2^UK Centre for Ecology and Hydrology, Wallingford, United Kingdom

**Keywords:** mathematical modeling, COVID-19, lockdown, health policy, Circuit Breaker (CB) interruption

## Introduction

This opinion submission is based upon the modeling framework previously published in Frontiers in Public Health: “*How and When to End the COVID-19 Lockdown: An Optimization Approach”* (1).

To curb an initial rapid increase in COVID-19 cases, the UK entered a nationwide “lockdown” on March 23rd 2020, where people were instructed to stay in their homes except for essential journeys. This generally proved successful, decreasing the *R*_0_ value to approximately 0.81 (2) during this period. The UK has since begun to ease these lockdown measures, with businesses being allowed to operate and social gatherings permitted subject to certain limitations. However, infection numbers have since started to grow again, with the UK government reporting on September 25th 2020 a new *R*_0_ value between 1.2 and 1.5 across the country (3).

With growing concern that a second wave of COVID-19 infections could place stress on the National Health Service (NHS) during the busy winter months (4), there has been discussion as to the potential for shorter, temporary, “circuit breaker” lockdowns (5). These lockdowns could be enacted with the intention to reduce *R*_0_ briefly, slowing transmission and delaying a peak in infections. Alternatively, lowered infections could be followed by revised social distancing measures to keep *R*_0_ below the critical value of 1. These “circuit breaker” measures are considered an undesirable last resort, with current limitations already causing an estimated 12.4% loss to the UK's GDP (6), and have been met with growing public displeasure. As such there is a need to investigate more formally what will be gained from the use of “circuit breaker” lockdowns.

In June 2020, we published a model to simulate the impact of multiple lockdown and subsequent release strategies upon the UK (1). It was concluded that a gradual easing of lockdown measures was vital to ensure that any increase in infection numbers could be observed, constrained, and appropriate action taken. The model considered two separate Susceptible-Exposed-Infected-Recovered (SEIR) compartmental cascades, one for those in a state of lockdown, and one for those who were not, so as to simulate the need for shielding certain areas or sub-populations. We have now used this model framework to simulate the effect of a “circuit breaker” lockdown, and to assess the impact of such a lockdown on the infection dynamics.

### Circuit Breaker Modeling

We set the initial states of the system in accordance with the latest reports from the Real-time Assessment of Community Transmission (REACT) programme. They found approximately 0.089% of the population to currently be infected as of September 7th 2020 (7), and 3.6 million people in the UK are estimated to have contracted COVID-19 as of the end of June 2020 (8). Adding to this the new estimated cases (9), we assumed an initial recovered population of 4 million. The transmission rate (β; infected people per day from an infected individual) of the disease was reduced to 0.35 to represent the impact of continued social distancing measures, to reflect the reported *R*_0_ value of approximately 1.6 (3, 7). Lockdown measures were then assumed to halve transmission, reducing *R*_0_ to approximately 0.8 as reported in Grant (2). All other model parameters were kept as reported in the original study, including a fraction of “key workers” who would not be under circuit breaker restrictions. All code used in this assessment is provided at osf.io/tfcdw/.

**Figure 1:**
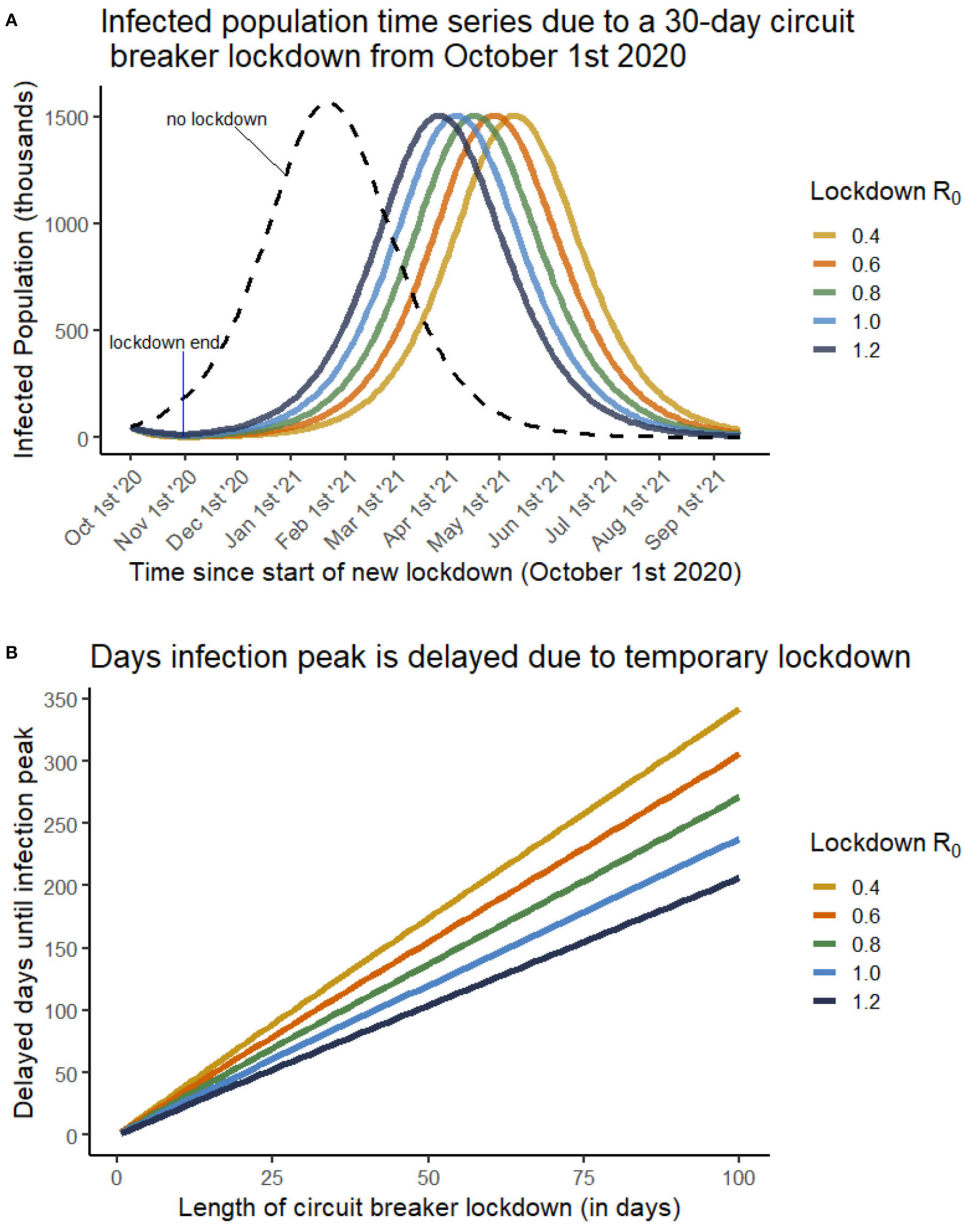
**(A)** The infected population through time if a 30-day circuit breaker lockdown is enacted from October 1st 2020. Each curve is for a different *R*_0_ value during the lockdown phase, as given in the legend. Once lockdown is ended, an *R*_0_ of 1.6 is considered. The dashed line depicts a scenario where no circuit breaker lockdown is enacted, and an infection peak is reached in late January 2021. **(B)** The number of days that infection peak is delayed by (y-axis) as a result of a temporary lockdown of variable duration (x-axis). Each curve is for a different *R*_0_ value during the lockdown phase, as given in the legend. Multiple lockdown effectiveness are considered, including cases where the lockdown does not succeed in lowering *R*_0_ below 1.

Without lockdown (i.e., no “circuit breaker”), our model reports an infection peak reached in 114 days (late January 2021). We considered the scenario where, from an initial time *t* = 0 corresponding to October 1st 2020, the UK was entered into a lockdown of variable duration. It was then recorded how many days from initialization it would take for the infection peak to occur as a result of an initial lockdown (Figure 1).

Figure 1A shows that lockdowns have very little impact on the magnitude of the infection peak. This is due to very few new infections occurring within a lockdown, meaning that roughly the same susceptible population is exposed following the end of lockdown. The peak is delayed due to a vastly reduced infected population under a stricter (or longer) lockdown, requiring substantially more time for the virus to re-establish itself within the population.

We see from Figure 1B that a near-linear relationship is always observed between duration of circuit-breaker lockdown and the delay of infection peak. In the case of an especially strict lockdown (*R*_0_ = 0.4) a 20-day circuit-breaker would result in moving the infection peak back roughly 75 days (to early April 2021), however in a weakly effective lockdown (*R*_0_ = 1.2), the 20 day circuit breaker would only delay the peak by roughly 40 days (to late February 2021). In each case, however, a benefit is still observed, suggesting that a temporary lockdown, of even low effectiveness, would be successful in delaying a second wave of COVID infections, providing a buffer to health services from simultaneously dealing with multiple, severe winter infections.

### Discussion

These results support the implementation of circuit breaker lockdowns as effective strategies to delay infection peaks, however they also argue that little impact will be seen in the total infection load and infection peak. As such, we advise policy makers to consider multiple factors when ultimately deciding upon resorting to such restrictions. Even in our simulations with only modest reductions in *R*_0_, the significant changes to dynamics will likely have considerable impact on the timings associated with other common upper respiratory infections such as the seasonal flu. Focusing on disrupting infection peaks will prevent overburdening health services through the next few months. It is also important to consider how a circuit-breaker is announced, and which measures are implemented before its commencement. For instance, during the days before implementation, there may be alterations to behavior (e.g., additional levels of shopping for essentials and socializing), bringing people into closer contact than may have otherwise happened.

On October 23rd 2020, Wales began a 17-day “fire breaker” lockdown, while the rest of the UK has implemented a tiered system of restrictions depending on infection levels within individual regions. Should this circuit breaker lockdown show significant effectiveness at reducing *R*_0_ in Wales, we would argue that central and other devolved administrations in the UK should similarly consider the use of circuit breaker lockdowns; implementation of these lockdowns needs to be predicated on critical planning for additional public health measures that will be available after the lockdown period, such as pharmaceutical (e.g., vaccines), or further non-pharmaceutical (e.g., altered social distancing) interventions.

## Author Contributions

TR built the model and generated the figure. All authors contributed to writing the manuscript.

## Conflict of Interest

The authors declare that the research was conducted in the absence of any commercial or financial relationships that could be construed as a potential conflict of interest.
